# Supplementary material for: The use of artificial substrate units to improve inventories of cryptic crustacean species on Caribbean coral reefs
Source: PeerJ. 2020 Nov 23;8:e10389. doi: 10.7717/peerj.10389 (PMC7690294; doi:10.7717/peerj.10389)
Supplement: Supplemental Information 1 — Number of individuals by species, families and orders of Crustacea retrieved from artificial substrate units (ASUs) and from coral rubble collected in situ in the Bonanza reef unit of the Puerto Morelos Reef National Park in 2013–2014. [file peerj-08-10389-s001.docx]

**Manuscript: The use of artificial substrate units to improve inventories of cryptic crustacean species on Caribbean coral reefs**

**Authors:** Luz Verónica Monroy-Velázquez^1^, Rosa E. Rodríguez-Martínez^1^, Paul Blanchon^1^, Fernando Alvarez^2^

**Supplementary table 1**

Number of individuals by species, families and orders of Crustacea retrieved from artificial substrate units (ASUs) and from coral rubble collected *in situ* in the Bonanza reef unit of the Puerto Morelos Reef National Park in 2013-2014.

| **Order** | **Family** | **Species** | **ASUs** | | | |  | **Coral Rubble** | | | |
| --- | --- | --- | --- | --- | --- | --- | --- | --- | --- | --- | --- |
|  |  |  | A | B | C | D |  | A | B | C | D |
| **Amphipoda** | Ampeliscidae | *Ampelisca abdita* | 1 |  |  |  |  |  |  |  |  |
|  |  | *Ampelisca bicarinata* |  |  |  |  |  |  |  |  | 2 |
|  |  | *Ampelisca* sp |  |  |  |  |  |  |  | 5 | 2 |
|  | Amphilochidae | *Hourstonius tortugae* |  |  |  | 2 |  |  |  |  |  |
|  | Ampithoidae | *Ampithoe ramondi* |  | 1 |  |  |  |  |  |  |  |
|  |  | *Ampithoe* sp |  |  |  |  |  | 1 |  |  | 4 |
|  |  | *Pseudoampithoides incurvaria* | 4 |  | 1 | 4 |  | 2 |  |  | 1 |
|  | Anamixidae | *Anamixis cavatura* |  | 1 | 1 | 1 |  |  |  |  |  |
|  | Aoridae | *Bemlo*s sp |  |  |  |  |  |  |  | 1 |  |
|  |  | *Globosolembos smithi* | 1 |  |  | 1 |  |  |  |  | 3 |
|  |  | *Lembos unifasciatus* | 1 |  |  |  |  |  |  |  |  |
|  | Bateidae | *Batea cuspidata* | 1 |  | 1 |  |  |  |  |  |  |
|  | Caprellidae | *Hemiproto wigleyi* |  |  |  | 1 |  |  | 2 | 1 |  |
|  | Chevaliidae | *Chevalia aviculae* | 3 | 6 |  | 3 |  | 29 | 27 | 2 | 2 |
|  | Colomastigidae | *Colomastix janiceae* |  |  |  |  |  |  | 4 |  |  |
|  | Leucothoidae | *Leucothoe laurensi* |  |  |  |  |  |  |  | 3 |  |
|  |  | *Leucothoe spinicarpa* | 1 |  |  | 1 |  |  | 1 |  |  |
|  | Liljeborgiidae | *Idunella* sp |  |  |  |  |  |  | 1 |  |  |
|  |  | *Liljeborgia* sp |  | 1 |  |  |  |  | 4 | 5 |  |
|  | Lysianopsiidae | *Lysianopsis alba* | 4 |  | 1 | 4 |  |  |  |  | 1 |
|  |  | *Concarnes concavus* |  |  |  |  |  |  |  | 1 |  |
|  |  | *Hippomedon* sp |  |  |  |  |  | 1 |  |  |  |
|  | Maeridae | *Ceradocus sheardi* | 3 | 12 |  | 3 |  |  |  |  | 1 |
|  |  | *Dumosus* sp | 5 | 1 |  |  |  | 5 |  | 1 |  |
|  |  | *Elasmopus levis* |  |  |  | 17 |  |  |  |  | 2 |
|  |  | *Elasmopus rapax* | 36 | 6 |  | 6 |  | 13 | 6 | 4 |  |
|  |  | *Maerella* sp |  |  |  |  |  | 6 |  |  | 2 |
|  |  | *Quadrimaera quadrimana* | 1 |  |  |  |  |  |  |  |  |
|  | Megaluropidae | *Gibberosus myersi* |  |  |  |  |  |  |  |  | 3 |
|  | Melitidae | *Melita* sp |  |  |  |  |  | 1 |  |  |  |
|  |  | *Netamelita barnardi* | 1 |  |  |  |  | 1 | 1 | 2 |  |
|  |  | *Nuuanu muelleri* | 1 |  |  |  |  |  |  |  |  |
|  | Oedicerotidae | *Perioculodes cerasinus* | 1 |  |  |  |  | 1 |  |  |  |
|  | Photidae | *Pariphinotus seclusus* | 1 |  |  | 1 |  |  |  |  |  |
|  |  | *Gammaropsis atlantica* | 1 | 3 |  | 12 |  |  | 8 | 18 |  |
|  | Phoxocephalidae | *Eobrolgus* sp |  |  |  |  |  | 5 | 2 | 2 | 2 |
|  |  | *Metarphinia floridana* | 1 |  |  |  |  |  |  |  | 2 |
|  | Synopiidae | *Synopia* sp |  | 1 |  |  |  |  |  |  |  |
|  |  | *Synopia ultramarina* |  |  |  |  |  | 1 |  |  | 1 |
| **Cumacea** | Bodotriidae | Bodotriidae A |  |  |  |  |  |  |  | 1 |  |
|  |  | Mancomunatinae |  |  |  | 1 |  |  |  |  |  |
|  |  | *Vaunthompsonia floridana* |  |  |  |  |  | 1 |  |  | 1 |
|  |  | *Vaunthompsonia minor* |  |  |  |  |  |  |  | 1 | 1 |
|  | Leuconidae | *Leucon* sp |  |  |  | 1 |  |  | 1 |  |  |
|  | Nannastacidae | *Campylaspis heardi* |  |  |  |  |  |  | 1 |  |  |
|  |  | *Cubanocuma gutzi* |  |  |  |  |  |  |  |  | 1 |
|  |  | *Cumella clavicauda* |  |  |  |  |  |  | 1 | 1 |  |
|  |  | *Cumella garrityi* |  |  |  |  |  |  |  | 1 | 1 |
|  |  | *Cumella goesi* |  |  |  |  |  | 1 |  |  |  |
|  |  | *Cumella gomoiui* |  |  |  |  |  |  |  |  | 1 |
|  |  | *Cumella muriarui* |  |  |  |  |  | 1 |  |  |  |
|  |  | *Cumella ruetzleri* |  |  |  |  |  |  |  | 1 |  |
|  |  | *Cumella* sp G | 1 |  | 1 |  |  |  | 1 |  |  |
|  |  | *Cumella vicina* |  |  |  |  |  | 3 | 1 |  |  |
|  |  | *Schizotrema agglutinanta* |  |  | 2 | 5 |  |  |  |  |  |
| **Decapoda** | Alpheidae | *Alpheus amblyonyx* | 2 |  | 2 | 1 |  | 2 | 1 |  | 1 |
|  |  | *Alpheus armatus* |  |  |  | 2 |  |  |  |  | 1 |
|  |  | *Alpheus barbadensis* | 1 | 1 |  |  |  |  |  |  | 1 |
|  |  | *Alpheus bouvieri* |  |  |  |  |  | 1 |  |  |  |
|  |  | *Alpheus cylindricus* | 2 |  |  |  |  | 1 | 1 |  | 2 |
|  |  | *Alpheus floridanus* |  |  |  | 1 |  |  |  |  | 1 |
|  |  | *Alpheus formosus* | 1 |  | 1 |  |  | 1 |  | 1 |  |
|  |  | *Alpheus malleator* |  |  |  |  |  | 2 |  |  | 1 |
|  |  | *Alpheus normanni* | 1 |  |  |  |  | 1 |  |  |  |
|  |  | *Alpheus paracrinitus* |  |  |  |  |  |  |  |  | 1 |
|  |  | *Alpheus* sp | 11 |  | 1 | 6 |  |  | 1 | 1 | 2 |
|  |  | *Automate dolichognatha* | 1 |  |  |  |  |  | 1 |  |  |
|  |  | *Metalpheus rostratipes* | 1 |  |  |  |  |  | 2 |  |  |
|  |  | *Rostronia* sp |  |  |  |  |  | 1 |  |  |  |
|  |  | *Salmoneus* sp | 1 |  |  |  |  |  |  |  |  |
|  |  | *Synalpheus anasimus* |  |  |  |  |  | 2 |  |  |  |
|  | Axiidae | Axiidae |  |  |  |  |  | 1 |  |  |  |
|  | Diogenidae | *Calcinus tibicen* |  |  |  | 1 |  |  |  |  |  |
|  |  | *Clibabarius tricolor* |  |  |  | 1 |  |  |  |  |  |
|  |  | Diogenidae | 1 |  |  | 1 |  |  |  |  |  |
|  |  | ***Paguristes hernancortezi*** |  | 1 |  |  |  |  |  |  | 1 |
|  |  | *Paguristes* sp | 2 |  | 1 |  |  |  |  | 1 |  |
|  |  | *Petrochirus diogenes* |  | 1 |  |  |  |  |  |  |  |
|  | Hippolytidae | *Hippolyte curacaoensis* | 1 |  |  |  |  |  |  |  |  |
|  |  | *Lysmata intermedia* |  |  | 2 |  |  |  |  |  |  |
|  |  | *Lysmata* sp | 1 |  |  |  |  |  |  |  |  |
|  | Mithracidae | *Hemus cristulipes* | 1 |  |  |  |  | 3 |  |  |  |
|  |  | *Hemus* sp |  |  | 1 |  |  |  |  |  |  |
|  |  | *Mithraculus coryphe* | 6 | 6 | 3 | 5 |  |  | 1 | 5 | 8 |
|  |  | *Mithraculus forceps* | 7 | 3 |  |  |  |  | 5 |  |  |
|  |  | *Mithraculus sculptus* | 17 | 23 |  |  |  |  |  |  |  |
|  |  | *Mithrax* sp | 11 | 2 | 3 | 3 |  |  | 2 | 1 |  |
|  |  | *Omalacantha antillensis* |  | 3 | 2 | 1 |  |  |  |  | 1 |
|  |  | *Pitho aculeatha* | 1 |  |  | 2 |  |  |  |  |  |
|  |  | *Pitho lherminieri* |  |  |  |  |  | 2 | 1 |  | 2 |
|  |  | *Pitho mirabilis* |  | 5 |  |  |  |  |  |  |  |
|  |  | *Pitho* sp | 1 |  |  | 4 |  |  |  |  |  |
|  | Paguridae | Paguridae A |  |  |  | 1 |  |  |  |  |  |
|  |  | *Pagurus annulipes* | 1 |  |  |  |  |  |  |  |  |
|  | Palaemonidae | *Cuapetes americanus* | 2 |  | 1 |  |  |  |  |  | 1 |
|  |  | Palaemonidae |  |  |  | 1 |  |  |  | 1 | 1 |
|  |  | *Periclimenaeus ascidarum* |  |  |  | 1 |  |  | 3 |  |  |
|  |  | *Periclimenes* sp | 1 |  |  |  |  |  |  |  | 2 |
|  | Penaeidae | *Metapenaeopsis martinella* |  |  |  |  |  |  |  |  | 2 |
|  |  | Penaeidae |  |  |  |  |  |  |  |  | 1 |
|  | Pilumnidae | *Pilumnus longleyi* |  | 2 |  |  |  |  |  |  |  |
|  | Porcellanidae | *Petrolisthes galathinus* | 1 | 2 | 2 | 1 |  |  |  |  |  |
|  | Processidae | *Ambidexter symmetricus* |  |  | 1 |  |  |  |  |  |  |
|  |  | ***Processa profunda*** | 1 |  |  |  |  | 1 |  |  |  |
|  |  | ***Processa riveroi*** | 1 |  |  |  |  |  |  |  | 1 |
|  |  | *Processa* sp | 1 |  |  |  |  |  |  |  |  |
|  | Spongicolidae | *Microprosthema semilaeve* |  |  |  | 1 |  |  |  |  |  |
|  | Thoridae | *Thor floridanus* | 1 |  |  |  |  |  |  |  |  |
|  |  | *Thor* sp | 3 |  |  |  |  |  |  |  |  |
|  | Upogebiidae | Upogebiidae sp |  |  | 1 |  |  | 1 |  |  |  |
|  | Xanthidae | *Williamstimpsonia denticulatus* |  |  |  |  |  |  |  |  | 1 |
|  |  | Xanthidae |  | 1 |  | 1 |  |  |  |  |  |
| **Isopoda** | Anthuridea | *Amakusanthura magnifica* | 5 |  |  |  |  | 6 | 1 | 3 |  |
|  |  | *Amakusanthura signata* |  | 2 |  | 2 |  |  |  | 2 | 1 |
|  |  | *Apanthura cracenta* | 3 | 1 | 1 |  |  | 7 | 5 |  | 2 |
|  |  | *Cortezura confixa* |  |  |  |  |  |  |  | 1 |  |
|  |  | *Mesanthura bivittata* | 1 | 1 |  | 1 |  |  |  |  |  |
|  |  | *Mesanthura fasciata* |  | 3 |  |  |  |  |  |  |  |
|  |  | *Mesanthura hopkinsi* |  |  |  |  |  |  |  | 2 |  |
|  |  | *Mesanthura paucidens* |  |  |  |  |  | 1 |  |  |  |
|  |  | *Mesanthura pulchra* |  |  | 1 |  |  |  |  | 1 |  |
|  |  | *Mesanthura* sp |  |  |  |  |  | 1 |  |  |  |
|  |  | *Pendanthura hendleri* |  |  |  | 1 |  | 18 |  | 25 | 1 |
|  |  | *Pendanthura* sp |  |  |  |  |  | 1 |  | 24 |  |
|  |  | Anthuridea |  |  |  |  |  | 1 |  |  |  |
|  | Cirolanidae | *Calyptolana hancocki* | 1 |  |  |  |  |  |  |  |  |
|  |  | *Cirolana parva* | 7 | 39 | 1 | 43 |  | 19 | 4 |  | 46 |
|  |  | *Eurydice convexa* |  |  |  |  |  |  | 1 |  | 1 |
|  |  | *Metacirolana agaricicola* |  |  |  |  |  |  |  | 1 |  |
|  |  | *Metacirolana halia* | 3 | 6 |  | 2 |  | 19 | 2 |  | 1 |
|  |  | *Neocirolana obtruncata* |  |  |  |  |  |  | 3 |  |  |
|  | Corallanidae | *Excorallana antillensis* |  |  |  |  |  | 2 |  |  | 3 |
|  | Gnathiidae | *Gnathia beethoveni* |  | 1 |  |  |  |  |  |  |  |
|  |  | *Gnathia magdalensis* | 12 | 6 |  | 3 |  | 1 |  | 3 |  |
|  |  | *Gnathia puertoricensis* | 4 | 3 | 1 | 4 |  | 10 | 3 | 13 |  |
|  |  | *Gnathia* sp | 19 | 5 |  | 10 |  | 38 | 13 | 34 | 27 |
|  |  | *Gnathia vellosa* |  |  |  | 3 |  | 3 |  | 5 | 1 |
|  |  | *Gnathia virginalis* |  | 4 |  |  |  | 23 | 1 | 8 |  |
|  |  | *Gnathostenetroides pugio* |  |  |  |  |  | 7 |  | 1 |  |
|  | Janiridae | *Carpias algicola* |  | 1 |  | 1 |  |  | 1 | 2 | 2 |
|  |  | *Carpias triton* |  |  |  |  |  | 1 |  |  |  |
|  | Joeropsididae | *Joeropsis bifasciatum* | 1 |  | 1 | 1 |  | 2 | 1 |  |  |
|  |  | *Joeropsis personatus* |  |  |  |  |  |  |  | 1 |  |
|  | Leptanthuridae | *Accalathura crenulata* | 2 |  |  |  |  | 2 |  |  |  |
|  | Limnoriidae | *Limnoria platycauda* |  |  |  |  |  |  |  |  | 1 |
|  | Munnidae | *Uromunna reynoldsi* |  |  |  | 1 |  |  |  |  |  |
|  | Paranthuridae | *Colanthura* sp |  |  |  |  |  |  |  | 1 |  |
|  |  | *Paranthura floridensis* |  | 2 | 1 |  |  |  |  |  |  |
|  | Sphaeromatidae | *Cymodoce ruetzleri* | 1 | 13 |  |  |  | 4 |  |  | 1 |
|  |  | *Cymodoce* sp | 2 |  |  |  |  |  |  |  |  |
|  |  | *Dicerceis kensleyi* |  |  |  |  |  | 1 |  |  |  |
|  |  | Dynameninae A |  |  |  |  |  | 3 |  |  |  |
|  |  | *Geocerceis barbarae* |  | 11 |  | 1 |  | 1 |  |  |  |
|  |  | *Paracerceis caudata* | 2 | 13 |  |  |  | 2 |  | 2 |  |
|  | Stenetriidae | *Hansenium stebbingi* | 5 |  |  | 3 |  | 6 |  |  |  |
|  |  | *Lyocoryphe minocule* |  |  |  | 1 |  |  | 2 | 2 |  |
|  |  | *Machatrium spathulicarpus* |  |  |  |  |  |  |  | 1 |  |
|  |  | *Stenetrium bowmani* |  |  |  | 4 |  |  |  |  |  |
|  |  | *Stenetrium serratum* |  |  |  |  |  |  | 2 | 1 |  |
|  |  | *Stenobermuda* sp | 1 |  |  |  |  |  |  |  |  |
| **Tanaidacea** | Apseudidae | *Apseudes orghidani* |  |  |  |  |  | 4 |  |  |  |
|  |  | *Apseudes* sp A | 15 | 2 | 2 | 22 |  | 193 | 22 | 70 | 3 |
|  |  | *Paradoxapseudes bermudeus* |  |  |  | 1 |  | 1 | 1 | 8 | 1 |
|  | Kalliapseudidae | *Mesokalliapseudes bahamensis* |  |  |  |  |  |  |  | 1 |  |
|  |  | *Psammokalliapseudes granulosus* | |  |  |  |  | 1 |  |  | 1 |
|  | Leptocheliidae | *Alloleptochelia longimana* |  |  |  |  |  |  |  | 3 |  |
|  |  | *Chondrochelia dubia* | 24 | 21 | 9 | 93 |  | 58 | 15 | 63 | 26 |
|  |  | *Hargeria rapax* |  |  |  |  |  | 5 |  | 3 |  |
|  |  | *Pseudoleptochelia* sp A | 14 | 4 |  | 5 |  | 63 | 58 | 76 | 3 |
|  | Metapseudidae | *Apseudomorpha* sp A |  |  |  | 1 |  | 26 |  | 11 |  |
|  |  | *Pseudoapseudomorpha* sp A |  |  |  |  |  |  | 2 | 1 |  |
|  | Nototanaidae | *Nototanais* sp |  |  | 2 |  |  | 36 |  |  | 1 |
|  | Pagurapseudidae | *Pagurotanais bouryi* |  |  |  |  |  |  | 1 |  |  |
|  | Parapseudidae | *Dyscapseudes* sp |  |  |  | 2 |  |  |  |  |  |
|  |  | *Parapseudes* sp A | 2 |  |  | 1 |  | 6 | 5 |  | 1 |
|  | Paratanaidae | *Paratanais* sp A | 4 |  |  | 2 |  | 205 | 2 | 65 |  |
|  | Tanaididae | *Sinelobus stanfordi* | 8 |  |  | 7 |  | 35 | 13 | 11 | 1 |
|  |  | *Zeuxo kurilensis* | 1 | 2 |  |  |  | 4 |  | 35 |  |
